# Clinical impact of soluble Neuropilin-1 in ovarian cancer patients and its association with its circulating ligands of the HGF/c-MET axis

**DOI:** 10.3389/fonc.2022.974885

**Published:** 2022-10-21

**Authors:** Daniel Martin Klotz, Jan Dominik Kuhlmann, Theresa Link, Maren Goeckenjan, Lorenz C. Hofbauer, Andy Göbel, Tilman D. Rachner, Pauline Wimberger

**Affiliations:** ^1^ Department of Gynecology and Obstetrics, Medical Faculty and University Hospital Carl Gustav Carus, Technische Universität Dresden, Dresden, Germany; ^2^ German Cancer Consortium (DKTK), partner site Dresden and German Cancer Research Center (DKFZ), Heidelberg, Germany; ^3^ National Center for Tumor Diseases (NCT), Dresden, Germany: German Cancer Research Center (DKFZ), Heidelberg, Germany; Faculty of Medicine and University Hospital Carl Gustav Carus, Technische Universität Dresden, Dresden, Germany; Helmholtz-Zentrum Dresden - Rossendorf (HZDR), Dresden, Germany; ^4^ Division of Endocrinology, Diabetes, and Bone Diseases, Department of Medicine III, Technische Universität Dresden, Dresden, Germany

**Keywords:** ovarian cancer, soluble neuropilin-1, prognosis, blood-based biomarker, retrospective analysis, HGF, c-MET

## Abstract

**Background:**

Neuropilin (NRP) is a transmembrane protein, which has been shown to be a pro-angiogenic mediator and implicated as a potential driver of cancer progression. NRP-1 up-regulation in ovarian cancer tissue predicts poor prognosis. However, the clinical relevance of the soluble form of NRP-1 (sNRP-1) as a circulating biomarker in ovarian cancer patients is unknown.

**Methods/patients cohort:**

sNRP-1 levels were quantified in a cohort of 88 clinically documented ovarian cancer patients by a commercially available sNRP-1 enzyme-linked immunosorbent assay (ELISA) kit (Biomedica, Vienna, Austria). Patients (81.8% with FIGOIII/IV) received primary cytoreductive surgery with the aim of macroscopic complete resection (achieved in 55.7% of patients) and the recommendation of adjuvant chemotherapy in line with national guidelines.

**Results:**

Higher levels of sNRP-1 reflected more advanced disease (FIGO III/IV) and indicated a trend towards suboptimal surgical outcome, i.e. any residual tumor. sNRP-1 was neither related to the patients’ age nor the *BRCA1/2* mutational status. Patients with higher sNRP-1 levels at primary diagnosis had a significantly reduced progression-free survival (PFS) (HR = 0.541, 95%CI: 0.304 - 0.963; p = 0.037) and overall survival (OS) (HR = 0.459, 95%CI: 0.225 - 0.936; p = 0.032). Principal component analysis showed that sNRP-1 levels were unrelated to the circulating hepatocyte growth factor (HGF) and the soluble ectodomain of its receptor the tyrosine kinase mesenchymal–epithelial transition (c-MET), suggesting that there is no proportional serological concentration gradient of soluble components of the NRP-1/HGF/c-MET signaling axis.

**Conclusions:**

In line with the previously shown tissue-based prognostic role, we demonstrated for the first time that sNRP-1 can also act as a readily accessible, prognostic biomarker in the circulation of patients with ovarian cancer at primary diagnosis. Given its known role in angiogenesis and conferring resistance to the poly ADP-ribose polymerase (PARP) inhibitor olaparib *in vitro*, our results encourage more detailed investigation into sNRP-1 as a potential predictive biomarker for bevacizumab and/or PARP-inhibitor treatment.

## Introduction

Ovarian cancer is the leading cause of death among patients with gynecological malignancies and more than 70% of patients are diagnosed with advanced disease (1). The most important prognostic factor is the postoperative residual tumor burden (1, 2). The cornerstone of standard first-line treatment of advanced ovarian cancer involves surgical debulking, aimed at macroscopically complete tumor resection, followed by platinum/paclitaxel-based chemotherapy and maintenance treatment with the anti-angiogenic monoclonal antibody bevacizumab (3–6). More recently, in patients with ovarian cancer harboring homologous repair deficiency (HRD), defined by either the presence of a germline or somatic pathogenic breast cancer gene (*BRCA) 1/2* mutation and/or genomic instability, a combination of bevacizumab with the Poly ADP-ribose polymerase (PARP) inhibitor olaparib has been approved as maintenance therapy after response to first-line platinum-based chemotherapy (7). Likewise, the PARP inhibitor (PARPi) niraparib has been approved as sole maintenance therapy (without bevacizumab) after response to first-line treatment, independently of the HRD status (8). This very recent milestone of biomarker-guided, first-line PARPi treatment has been based on the knowledge that ovarian cancer with *BRCA1/2* mutations comprises a molecular Achilles’ Heel that can be exploited by targeting HRD (9). Hence, treatment with PARPi led to a markedly improved progression-free survival in patients with HR-deficient ovarian cancer (7, 8).

Despite these therapeutic advances, many patients with ovarian cancer still face a poor overall prognosis (2, 10). Given this clinical challenge, the identification of novel blood-based predictive and/or prognostic biomarkers is of high clinical significance. This would drive personalized treatment of ovarian cancer patients and guide future drug target identification.

Neuropilin (NRP) is a 120-140 kDa type I transmembrane protein, which is actively involved in a variety of physiological processes, such as cardiovascular development, activity of regulatory T cells (Tregs) and neuronal guidance (11–13). Two neuropilin homologues have been identified in vertebrates, referred to as NRP-1 and NRP-2 (12). NRP-1 is strongly expressed in the tumor vasculature and is a high-affinity co-receptor for a number of vascular endothelial growth factor (VEGF) isoforms, particularly VEGF_165_, resulting in an increased affinity of VEGF_165_ for the extracellular domain of VEGFR2 (12, 14, 15). Therefore, NRP-1 has been shown to be a pro-angiogenic mediator and implicated as a potential driver of metastatic cancer progression. Besides its interaction with VEGFR2, NRP-1 acts as a co-receptor for a number of other extracellular ligands, such as semaphorins, hepatocyte growth factor (HGF) and transforming growth factor beta (TGF-β) (13).

Preclinical studies have suggested that NRP-1 expression is up-regulated in ovarian cancer tissue and correlates with advanced FIGO stage and lymph node metastasis (16, 17). Moreover, NRP-1 expression was associated with epithelial to mesenchymal transition (EMT) markers (18) and PARPi resistance (19). It was proposed that high NRP-1 expression in the primary tumor predicts poor prognosis in ovarian cancer patients (16). Since a tissue-based biomarker is restricted to the histological analysis of cancerous tissue, the identification of blood-based biomarkers is of high clinical interest in ovarian cancer diagnostics biomarkers because they offer relatively easy and safe sampling for follow-up analysis and disease monitoring. This is particularly true because tissue samples of ovarian cancer are typically only obtained at primary cytoreductive surgery. In contrast, surgical treatment at first disease recurrence is clinically indicated and performed only in a specific subset of patients, i.e. in whome macroscopically complete tumor resection can be achieved (20). In addition to its transmembrane configuration, NRP-1 is also shed into circulation as soluble NRP-1 (sNRP-1), where it lacks the transmembrane and cytoplasmic domain. sNRP-1 is robustly detectable in human serum samples, as we have previously shown in patients with early breast cancer (21). We were able to demonstrate that breast cancer patients with low levels of sNRP-1 had a significantly better prognosis compared to patients with high levels of sNRP-1 (21). However, the clinical relevance of sNRP-1 and its potential prognostic value in patients with ovarian cancer is completely unknown.

The aim of this study was to profile sNRP-1 levels in serum samples of a comprehensive set of clinically documented ovarian cancer patients and to study its relation to patients’ clinicopathological parameters and its prognostic relevance. Moreover, we compared sNRP-1 levels with levels of selected soluble components of NRP-1 interaction partners, i.e. soluble HGF (sHGF) and the soluble ectodomain of the tyrosine kinase mesenchymal–epithelial transition (c-MET), referred to as soluble/serum c-MET (sMET).

## Patients and methods

### Patient characteristics and healthy controls

Patients were recruited at the Department of Gynecology and Obstetrics at the Carl Gustav Carus University of Dresden, Technische Universität Dresden, Germany. Overall, 88 patients with histologically confirmed primary epithelial ovarian cancer (primary diagnosis from 2013-2019, 81.8% with FIGOIII/IV) were included. Inclusion criteria were: primary cytoreductive surgery at our hospital with the aim of macroscopic complete resection and the recommendation of adjuvant platinum‐/paclitaxel-based chemotherapy in line with national guidelines. In the case of no contraindications, patients with a tumor stage of at least FIGO IIIb (50/72 patients, 69.4%) were additionally treated with the monoclonal antibody bevacizumab and enrollment in clinical trials was permitted. Exclusion criteria were: primary/neo-adjuvant chemotherapy, interval debulking surgery, treatment with hyperthermic intraperitoneal chemotherapy, benign disease or borderline tumors. Progression-free survival (PFS) and overall survival (OS) were calculated from the date of primary diagnosis (i.e. at the time of primary debulking surgery). 30 healthy women were also recruited. In order to be included in this study, these women must have had no past medical history of benign or malignant disease. The median age was 38 (range: 31 – 47 years). Written informed consent was obtained from all study participants and the study was approved by the Local Research Ethics Committee in Dresden (EK74032013). All study methodologies conformed to the standards set by the Declaration of Helsinki. The clinical data from the patients are summarized in **Table 1**. Tumor staging was documented according to the Fédération Internationale de Gynécologie et d’Obstétrique (FIGO) (22), revised in 2014 (23). Hence, the revised version was used for all patients who underwent primary surgery from 2014 onwards. In agreement with national recommendations, genetic testing was offered and performed, if patients consented (24, 25). Given the significant oncological implementation, BRCA status was analyzed in all patients from whom genetic testing had been documented. Germline *BRCA*1/2 mutational status was available in 39/88 patients. It is important to note that HRD analyses were not routinely tested outside of clinical trials at the time of primary diagnosis (2013-2019) in this retrospectively analyzed patient cohort.

**Table 1:** Patient characteristics.

Patients	N	88	
Age	median (range)	65 years	(23-82years) -
FIGO stage	1/11	16	18.2%
	III/IV	72	81.8%
Surgical debulking	residual disease	39	44.3%
	No residual tumor	49	55.7%
Histology	serous	78	88.6%
	non-serous	10	11.4%
Grading	high-grade (G3)	76	86.4%
	G1/G2	12	13.6%
BRCA1/2 mutational status	wtBRCA1/2	24	27.3%
	mBRCA1/2	15	17.0%
	unknown	49	55.7%
sNRP-1levels	median (range)	2.358 nmol/L(1.049-	- 5.126 nmol/L)
Progression-free survival	median (range)	30 months	(1 - 86 months)
	progression/death	49	55.7%
	no progression	39	44.3%
Overall survival	median (range)	42 months	(3 - 89 months)
	dead	33	37.5%
	alive	55	62.5%

### Serum preparation and detection of sNRP-1

Serum preparation from blood-samples obtained at primary diagnosis of ovarian cancer was performed, as described previously (26–28). Briefly, sample processing occurred within 1 h of blood drawing. After obtaining blood samples, they were incubated at room temperature (rt) for at least 30 min in order to allow complete blood coagulation. The cell-free serum fraction was obtained by centrifugation (8 min, 1800 g, rt) and was then immediately frozen at −80°C until further use. In order to compare pre-processing of control samples and patient samples were performed with the same protocol.

After complete thawing on ice, samples were immediately processed. The NRP-1 ELISA was performed as described previously (21). Briefly, 10 µl of the sample was used per well and the NRP-1 ELISA was conducted according to the manufacturer’s protocol (Biomedica, Vienna, Austria). The absorbance was measured immediately at 450 nm with reference at 630 nm.

### Statistical analysis

The statistical analysis was conducted with R, Version 3.6.2 and GraphPad Prism version 8.4.3 (GraphPad Software, La Jolla, CA, USA) as described previously (26–28), and listed in each figure legend. P‐values < 0.05 were considered statistically significant. The Hodges-Lehman estimate was used to determine the estimated differences (ED) of medians. Uni‐ and multivariate Cox proportional hazards model regression analyses were performed and hazard ratios (HRs) are indicated with 95% confidence intervals (CI). The median (2.358 nmol/L) has been used to stratify patients into sNRP-1 high (n = 44) and sNRP-1 low (n = 44), unless specified otherwise. The optimized cut off analysis was performed using maximally selected rank statistics (maxstat package). Kaplan–Meier analyses were performed with significance levels indicated by log-rank (Mantel-Cox) analysis and HRs (Mantel-Haenszel) are shown with 95%CI. The correlation between sNRP-1 levels with age or cancer antigen 125 (CA125) was assessed by non-parametric Spearman correlation. Correlation-based principal component analysis was performed, using Pearson correlation.

## Results

### Soluble sNRP-1 levels at primary diagnosis of ovarian cancer

**Figure 1:**
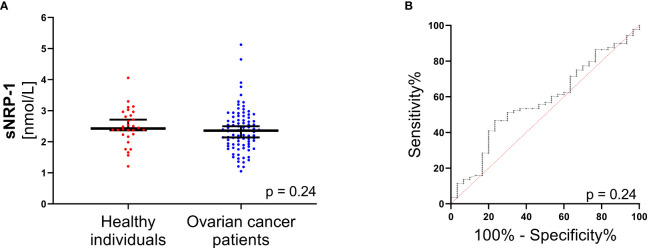
sNRP-1 levels in ovarian cancer at primary diagnosis. **(A)** Scatter plots comparing sNRP-1 levels in ovarian cancer patients (n = 88) and in healthy individuals (n = 30). The black horizontal lines indicate median sNRP-1 levels in each group, with error bars showing the 95%CI. P-value according to the non-parametric, two‐sided Mann-Whitney test. **(B)** Receiver operating characteristic (ROC) analysis to determine the diagnostic ability of sNRP-1 levels to distinguish between ovarian cancer patients (n = 88) and healthy controls (n = 30). The respective area under the curve (AUC) values and the 95%CIs are indicated.

We analyzed the sNRP-1 level in a comprehensive cohort of 88 clinically documented ovarian cancer patients at primary diagnosis and compared it to the level of healthy controls (n = 30). There was no significant difference between median sNRP-1 in ovarian cancer patients *vs.* healthy controls (estimated difference (ED) = -0.15, 95%CI: -0.39 - 0.12; p = 0.24; **Figure 1A**). This was supported by the receiver operating characteristic (ROC) analysis, which failed to show any discrimination between patients and healthy controls by sNRP-1 levels (p = 0.24; **Figure 1B**), meaning that sNRP-1 cannot be considered as a *bona fide* diagnostic marker without additional parameters.

### Correlation of sNRP-1 level with clinicopathological features of ovarian cancer

**Figure 2:**
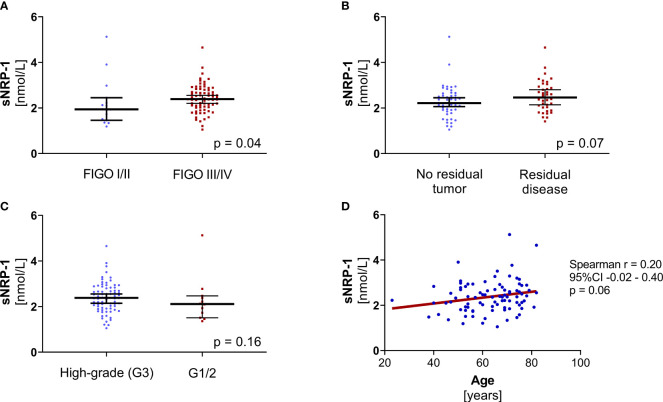
Association analyses of sNRP-1 with known clinical parameters. **(A)** The sNRP-1 levels of patients with advanced ovarian cancer (FIGO III/IV, n = 72) compared to patients with early-stage disease (FIGO I/II, n = 16), p = 0.04. **(B)** The sNRP-1 levels of patients (n = 39) with residual tumor compared to patients (n = 49) with no macroscopic tumor after cytoreductive surgery, p = 0.07. **(C)** The sNRP-1 levels of patients (n = 76) with high-grade ovarian cancer compared to patients (n = 12) with lower grading, p = 0.16. The black horizontal lines indicate the median sNRP-1 levels in each group with error bars, showing the 95%CI. P-values according to the non-parametric, two‐sided Mann-Whitney test. **(D)** The correlation of sNRP-1 and age is shown, using non-parametric Spearman correlation (n = 88, p = 0.06) with simple linear regression (red line).

Higher levels of sNRP-1 reflected more advanced disease, indicated by a higher FIGO stage (ED = 0.42, 95%CI: 0.04 - 0.70; p = 0.04; **Figure 2A**). Moreover, higher sNRP-1 levels at primary diagnosis showed a non-significant but numerical trend to be associated with suboptimal surgical outcome (ED = 0.26, 95%CI: -0.01 - 0.53; p = 0.07; **Figure 2B**). There was also neither a correlation between sNRP-1 levels between high-grade *vs.* lower grading (low-grade and moderately-differentiated) ovarian cancer (ED = -0.29, 95%CI: -0.66 - 0.12; p = 0.16; **Figure 2C**) nor the patients’ age (r = 0.20, 95%CI: -0.02 - 0.40; p = 0.06; **Figure 2D**).

The *BRCA*1/2 mutational status was available in 39 of 88 patients in our cohort (44.3%). Of those, 24/88 patients (27.3%) were *BRCA1/2* wild type (wt*BRCA1/2*), whereas in 15/88 patients (17.0%) a pathogenic *BRCA1/2* mutation (m*BRCA1/2*) had been detected. There was no significant difference in sNRP-1 levels between m*BRCA*1/2- *vs*. wt*BRCA1/2-*patients (ED = -0.01, 95%CI: -0.42 - 0.38; p=0.97; **Supplementary Figure 1A**). Information on CA125 at primary diagnosis was available in all patients (n = 88). We observed a correlation between sNRP-1 and CA125 (r = 0.22, 95%CI: 0.001 - 0.419; p = 0.04; **Supplementary Figure 1B**).

Taken together, sNRP-1 at primary diagnosis is unrelated to *BRCA1/2* mutational status, correlates with advanced disease and associates with surgical outcome by trend.

### Prognostic relevance of sNRP-1

**Figure 3:**
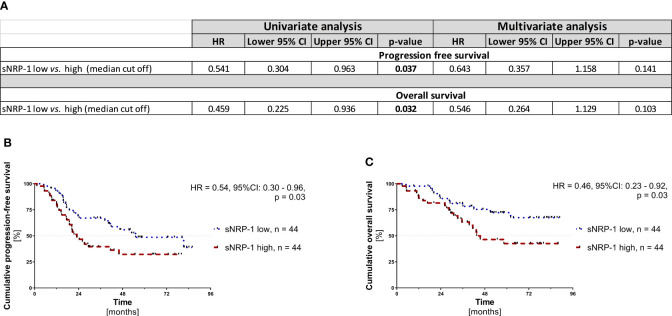
Prognostic relevance of sNRP-1. **(A)** Results from univariate and multivariate Cox proportional hazard regression model analyses of sNRP-1 low (n = 44) *vs*. sNRP-1 high (n = 44) are shown, including hazard ratio (HR) and 95%CIs and p-values. Kaplan-Meier analyses comparing **(B)** cumulative progression-free survival (PFS) and **(C)** cumulative overall survival (OS) of patients with ovarian cancer stratified as above. HR and 95%CI determined by Mantel Haenszel and p-value by log-rank (Mantel-Cox), as described in the methods section.

Using the median sNRP-1 level as a cut-off value, we stratified our study cohort into sNRP-1 high (>2.358 nmol/L) *vs*. sNRP-1 low (<2.358 nmol/L) patients and performed a Cox proportional hazards model regression and Kaplan-Meier analyses. We observed that higher sNRP-1 levels at primary diagnosis of ovarian cancer were associated with significantly reduced PFS (HR = 0.541, 95%CI: 0.304 - 0.963; p = 0.037) and OS (HR = 0.459, 95%CI: 0.225 - 0.936; p = 0.032) in the univariate but not multivariate analysis (**Figure 3A**). This was consistent with Kaplan-Meier analyses, indicating that higher sNRP-1 levels predict a significantly reduced PFS (HR = 0.54, 95%CI: 0.30 - 0.96; p = 0.03) and OS (HR = 0.46, 95%CI: 0.23 - 0.92; p = 0.03; **Figures 3B, C**). In the above analyses, we have used the median as cut off for grouping the patient into sNRP-1 high or sNRP-1 low. Another approach for dichotomizing a patient cohort with an optimized cut-off can be performed by maximally selected rank statistics. This resulted in the following cut offs: OS: >2.9805 nmol/L or PFS: > 2.3195 nmol/L. Using this optimized cut off as means to group our patient cohort into sNRP-1 high vs. sNRP-1 low, an even more pronounced prognostic relevance of sNRP-1 became evident in the Kaplan-Meier analysis (PFS: HR = 0.49, 95%CI: 0.28.-0.88; p = 0.02 and OS: HR = 0.12, 95%CI: 0.03 - 0.45; p = 0.002; **Supplementary Figures 2A, B**). It was also observed that higher sNRP-1 levels at primary diagnosis of ovarian cancer were associated with a significantly reduced PFS (HR = 0.491, 95%CI: 0.272 - 0.885; p = 0.018; **Supplementary Figure 2C**) in the univariate but not multivariate cox proportional hazards model regression analysis. Notably, Cox proportional hazards model regression analysis could not be performed for the OS analysis because the stratification using this optimized cut off did not meet the proportional hazards assumption.

This demonstrates that sNRP-1 can be considered as a blood-based prognostic biomarker in ovarian cancer patients. High levels of sNRP-1 indicate higher risk of disease recurrence and poor survival.

### Association of sNRP-1 with serum levels of HGF and the soluble ectodomain of c-MET

**Figure 4:**
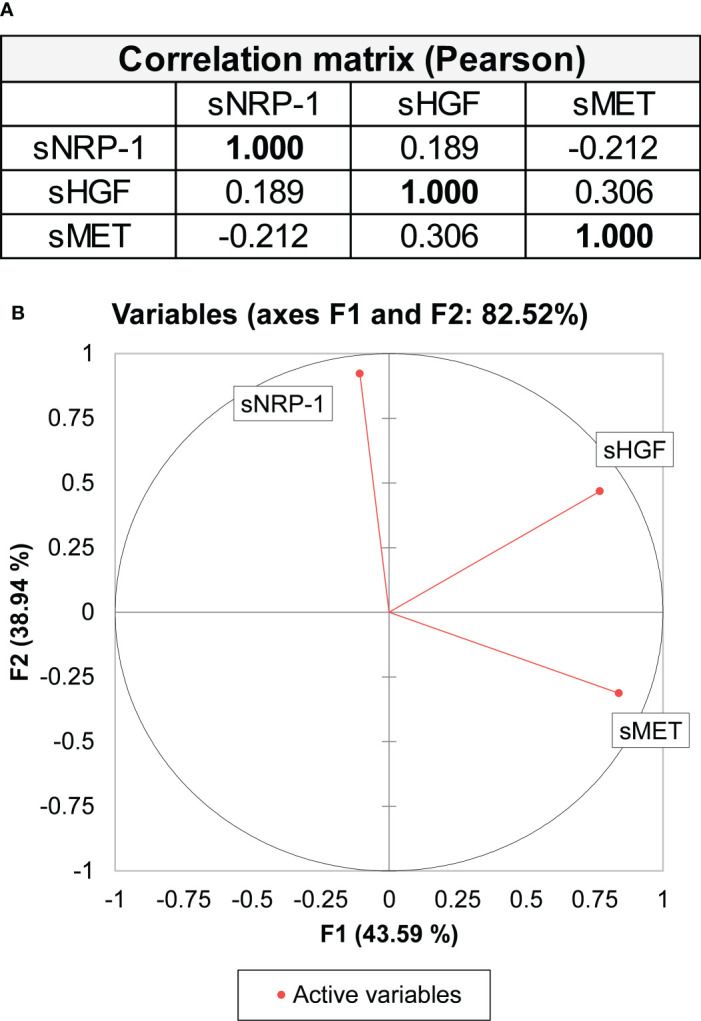
Principal component analysis using sNRP-1, sHGF and sMET levels. **(A)** A correlation matrix as a principal component analysis (Pearson) is shown with sNRP-1, soluble HGF (sHGF) and soluble ectodomain of c-MET (sMET). Values in bold are different from 0 with significance as p < 0.05. Biomarker levels were measured in blood samples of the same ovarian cancer patient at primary diagnosis. **(B)** Graphical representation of the principal component analysis of the three variables (sNRP-1, sHGF and sMET) contributing to 82.52% of the variability of the data set.

In addition to its interaction with VEGFR2, NRP-1 is a co-receptor for a number of other extracellular ligands, including c-MET and HGF (13, 29, 30). We hypothesized that there could be an association between the level of sNRP-1 and associated ligands in the blood of ovarian cancer patients. We took advantage of our previous studies on ovarian cancer, which demonstrated the prognostic relevance of both sHGF levels and the soluble ectodomain of its receptor c-MET (sMET) (26, 27). Corresponding data on sHGF and sMET levels were available in 35/88 and 26/88 of our patients from two previous studies of our group, respectively (26, 27). This number of matching samples allowed us to investigate whether there was a proportional serological concentration gradient of sNRP-1 and its functionally related proteins sHGF and sMET. We performed a principal component analysis, assessing all three serological biomarkers sNRP-1, sMET and sHGF. However, there was no significant correlation/clustering obtained by analyzing all three biomarkers (**Figures 4A, B**).

## Discussion

This is the first study that investigated the clinical relevance of sNRP-1 in blood samples of patients with ovarian cancer at primary diagnosis, demonstrating that high sNRP-1 indicates advanced disease and poor prognosis.

This is supported by an earlier study, which demonstrated that NRP-1 upregulation in ovarian cancer indicated poor prognosis when analysing tissue, gene and protein expression levels (16). Our findings complement the pro-tumorigenic effects of NRP-1 in cancer cells, such as modulating EMT, evasion of contact inhibition or promoting angiogenesis (12, 14, 31, 32).

However, the origin and function of sNRP-1 is still unclear. Firstly, the pool of sNRP-1 could be derived, at least partially, from cancer cells or the tumour microenvironment. If true, one would assume that more aggressive tumors may release more sNRP-1. This is consistent with our observation that higher sNRP-1 levels correlate with a poor prognosis and advanced disease. However, median sNRP-1 levels did not significantly differ between healthy women and patients with ovarian cancer (**Figure 1A**). This finding is consistent with reports showing no significant difference of sNRP-1 in patients with non-advanced breast cancer or malignant vocal lesions compared to healthy controls or patients with benign vocal cord lesions, respectively (22, 33).

The shedding rate may also influence sNRP-1 concentrations, which may differ by cancer type and could partially explain the above observations. Another circulating ovarian cancer biomarker (sMET) also offers prognostic relevance despite similar median levels in serum of patients and healthy controls (26). One can speculate whether the tumor microenvironment potentiates the effect of sNRP-1 once malignant transformation occurred. In a preliminary study, sNRP-1 levels in human serum ranged from a median of 4.62 nmol/L (range: 2.10 - 8.87 nmol/L) (34). Since the study did not disclose specific characteristics of study participants, one must speculate which factors contributed to sNRP-1 levels in these individuals.

Since both tissue and blood-based NRP-1 levels allow for prognostic stratification in ovarian cancer (16), further investigation should aim to investigate 1) the cellular processing of NRP-1, 2) its release from the tumor microenvironment in patients with ovarian cancer, and 3) determinants of its concentration in non-malignant physiological conditions.

Our exploratory study has certain limitations, i.e. the medium-sized patient cohort, a lack of comparison with tissue-based NRP-1 expression and the retrospective setting. Nonetheless, the strength of our study is that we can show prognostic relevance of our marker candidate in a well-documented patients cohort, considering all relevant clinicopathological parameters and including BRCA1/2 mutational status.

It is important to note that the present study refers to patients with a primary diagnosis of ovarian cancer from 2013-2019. At this time, the addition of the PARPi olaparib as maintenance treatment after response to first-line chemotherapy was restricted to patients with *BRCA1/2*-mutant advanced ovarian cancer. Only one patient with a germline *BRCA1* mutation received olaparib in our patient cohort as maintenance treatment following response to first-line chemotherapy. Given this is the first study describing a prognostic relevance of sNRP-1, it will be interesting to prospectively investigate the use of sNRP-1 in patients with HR-deficient ovarian cancer receiving maintenance therapy with bevacizumab and/or PARPi according to standard clinical practice (7, 8). Since NRP-1 promotes angiogenesis (35), previous studies have assessed whether it could predict response to bevacizumab at primary diagnosis. However, NRP-1 expression in ovarian cancer tissue failed to predict bevacizumab response in a retrospective analysis of the GOG-0218 clinical trial (36).

Interestingly, a previous study demonstrated a potential role of NRP-1 in conferring olaparib resistance *in vitro* (19). Both the pro-angiogenic activity of NRP-1 and its link to PARPi resistance would strongly suggest a potential use as a suitable auxiliary marker for predicting response to the combination of bevacizumab/olaparib in patients with ovarian cancer. This is of particular importance because PARPi treatment is expanding, resulting in an increasing number of patients with acquired (or primary) PARPi resistance in clinical practice. Furthermore, it would also be of clinical importance to determine the prognostic relevance of sNRP-1 in each subtype of ovarian cancer (37). Given the heterogeneous nature of ovarian cancer, this may also improve our understanding of sNRP-1 release and its correlation with tissue expression, if subtype-specific patterns are observed.

We have previously shown the use of sHGF and sMET as an independent prognostic biomarker in patients with ovarian cancer (26). HGF is a pleiotropic cytokine and a potent growth and pro-angiogenic factor that acts on its target cells by binding to the c-MET receptor. Moreover, HGF and c-MET also interacts with neuropilins (29, 38). However, we did not observe any correlation between sNRP-1, sHGF or sMET in a subset of corresponding patients’ serum samples, indicating that there may not be a proportional serological concentration gradient of sNRP-1 and circulating HGF and/or c-MET. Considering the broad spectrum of NRP-1 interacting ligands (39), a combined analysis of sNRP-1 and other functionally related proteins may still yield a biomarker signature that would enable additional prognostic or predictive information.

## Conclusion

We show for the first time, that NRP-1 is a blood-based prognostic biomarker, which could be easily implemented into routine clinical diagnostics of ovarian cancer. Our results encourage a prospective validation study to analyse whether sNRP-1 detection could be considered as an auxiliary predictive or prognostic tool in patients with ovarian cancer. This will be of future clinical relevance given its interaction with VEGF and conferring olaparib resistance *in vitro* (14, 19).

## Data availability statement

Upon reasonable requests, the raw data supporting the conclusions of this article will be made available by the authors, without undue reservation.

## Ethics statement

The studies involving human participants were reviewed and approved by ETHIKKOMMISSION AN DER TECHNISCHEN UNIVERSITÄT DRESDEN (EK74032013). The patients/participants provided their written informed consent to participate in this study.

## Author contributions

DMK, JDK, TDR, LCH and PW made substantial contributions to the conception and design of the study. DMK, JDK, TL, AG, MG and PW contributed to the experimental work or to the acquisition of clinical samples/data or to the analysis/interpretation of the results. DMK, JDK, TL, TDR, and PW were involved in drafting the manuscript, creating figures and/or revising the manuscript. All authors read and approved the manuscript in its final version.

## Funding

DMK was supported by the Else Kröner-Fresenius-Stiftung in the form of a clinician scientist program referred to as ‘phosphoproteome dynamics’ (Grant number 060_5217), and by the Central German Society for Gynecology and Obstetrics (Mitteldeutsche Gesellschaft für Frauenheilkunde und Geburtshilfe).

## Acknowledgments

The authors would like to thank Babett Heschel for her excellent technical assistance and Dr. M. Stevense (TU Dresden, Germany) for language editing.

## Conflict of interest

DMK has a patent application pending regarding the use of HGF as a prognostic biomarker in ovarian cancer.

The remaining authors declare that the research was conducted in the absence of any commercial or financial relationships that could be construed as a potential conflict of interest.

## Publisher’s note

All claims expressed in this article are solely those of the authors and do not necessarily represent those of their affiliated organizations, or those of the publisher, the editors and the reviewers. Any product that may be evaluated in this article, or claim that may be made by its manufacturer, is not guaranteed or endorsed by the publisher.

## Supplementary material

Supplementary Figure 1 Association of sNRP-1 with *BRCA1/2* mutational status and CA125. **(A)** Scatter plots with sNRP-1 levels of patients with known *BRCA1/2* mutational status are shown with *BRCA1/2* mutations (*mBRCA1/2*, n =15) and wild-type *BRCA1/2* status (*wtBRCA1/2*, n = 24), p = 0.97. **(B)** The correlation of sNRP-1 and log (CA125) is shown, using non-parametric Spearman correlation (n = 88, p = 0.04) with simple linear regression (red line).

Supplementary Figure 2 Prognostic information of sNRP-1 using an optimized cut-off. Kaplan-Meier analysis of patients with ovarian cancer comparing **(A)** cumulative progression-free survival according to sNRP-1 low (n = 42) and sNRP-1 high (n = 46), optimized cut-off PFS: 2.3195 nmol/L and **(B)** cumulative overall survival (OS) according to sNRP-1 low (n = 77) and sNRP-1 high (n = 11), optimized cut-off: 2,9805 nmol/L. HR and 95%CI determined by Mantel Haenszel and p-value by log-rank (Mantel-Cox), as described in the methods section. **(C)** Results from univariate and multivariate Cox proportional hazard regression model analyses for PFS of sNRP-1 low (n = 42) *vs*. sNRP-1 high (n = 46) are shown, including hazard ratio (HR) and 95%CIs and p-values. **(D)** Scatter plot is shown with sNRP-1 low (n = 42) and sNRP-1 high (n = 46) according to an optimized cut-off determined by maximally selected rank statistics for PFS, p < 0.0001. The black horizontal lines indicate the median sNRP-1 levels in each group with error bars, showing the 95%CI. P-value according to the non-parametric, two‐sided Mann-Whitney test. **(E)** Graphical representation of cut-off determination by maximally selected rank statistics.
