# Supplementary figures and images for: Clinical impact of soluble Neuropilin-1 in ovarian cancer patients and its association with its circulating ligands of the HGF/c-MET axis
Source: Front Oncol. 2022 Oct 21;12:974885. doi: 10.3389/fonc.2022.974885 (PMC9635484; doi:10.3389/fonc.2022.974885)

## Slide 1
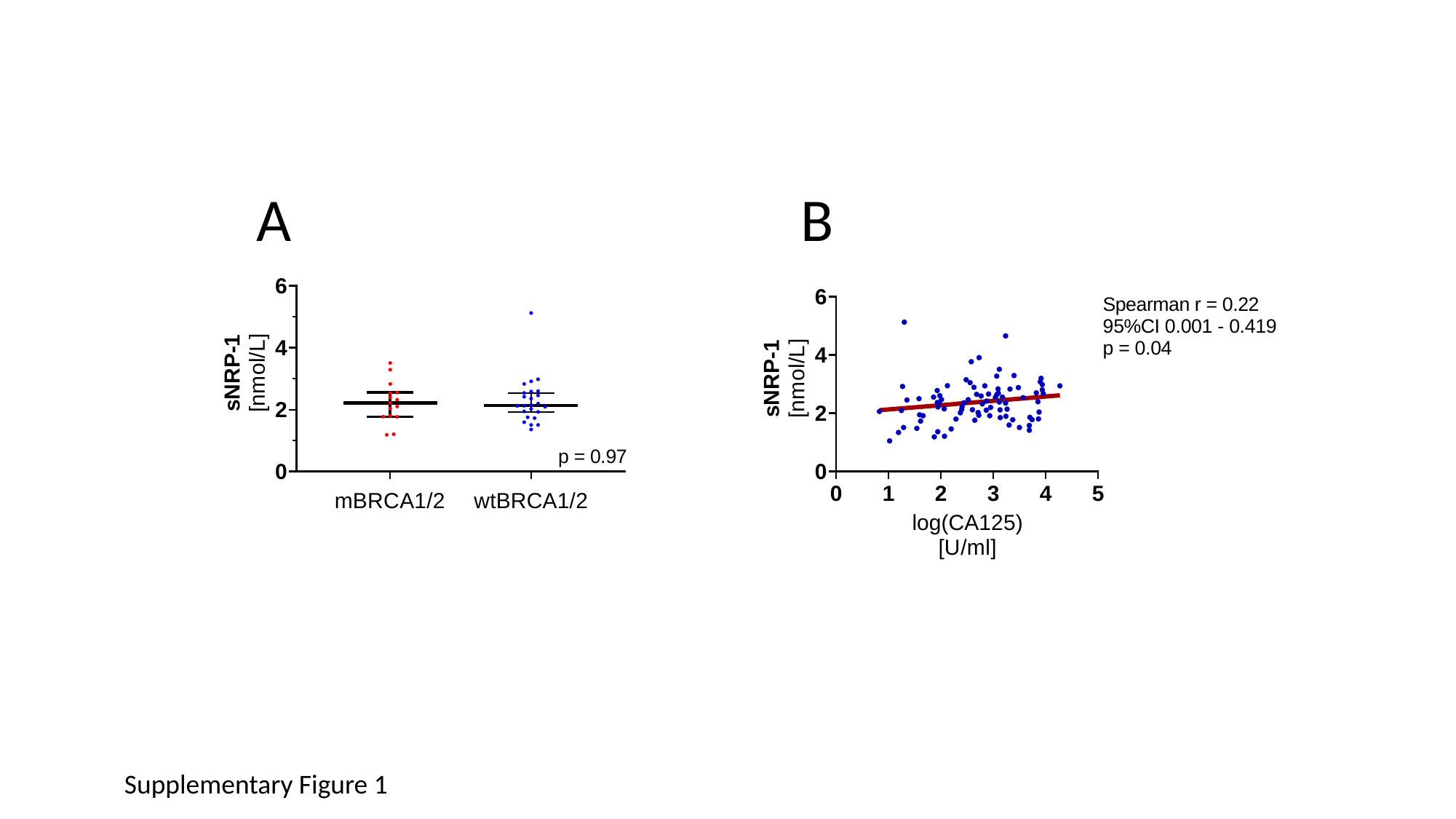

A
B
Supplementary Figure 1

## Slide 2
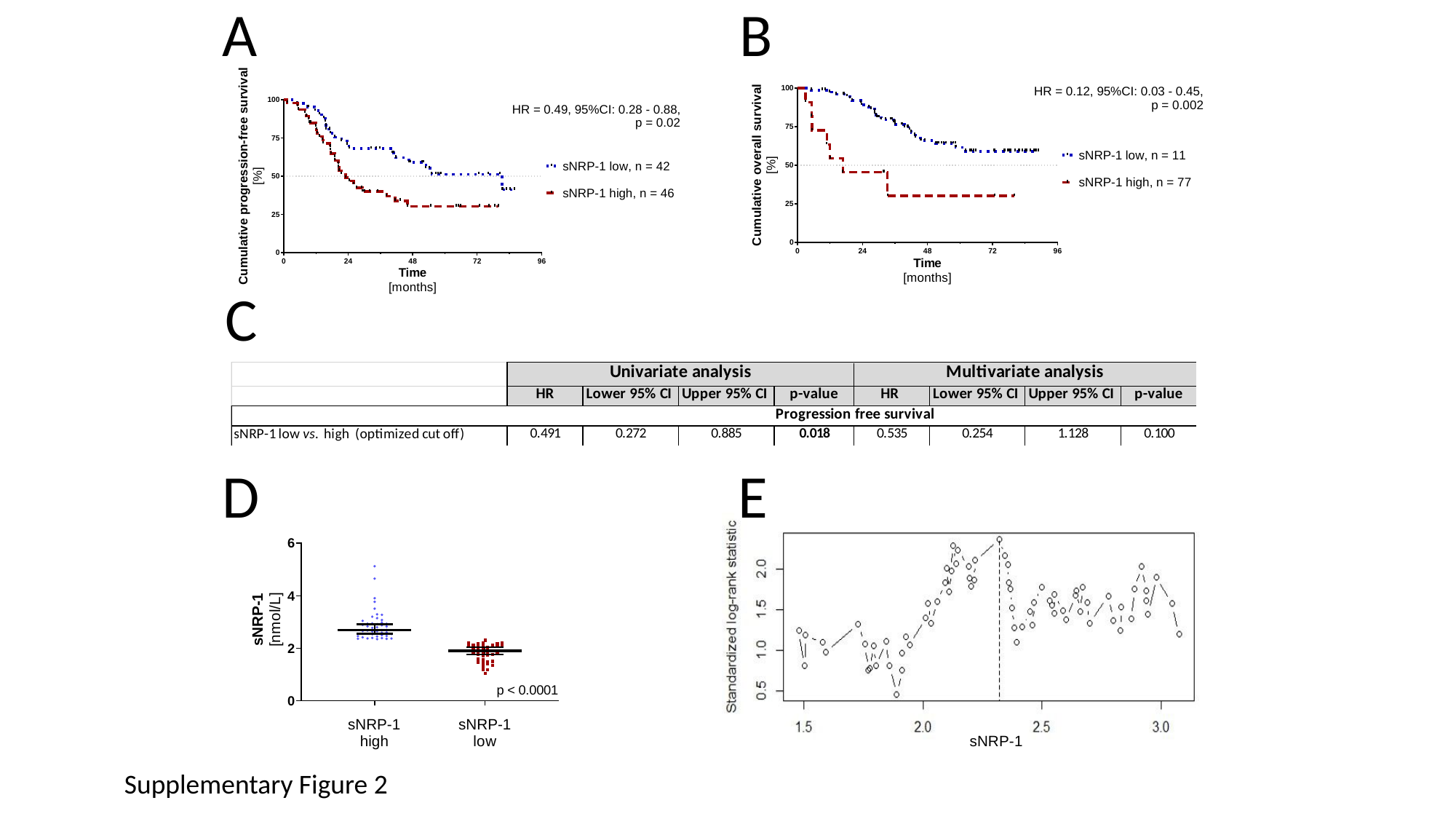

A
B
C
D
E
Supplementary Figure 2

Supplement: Supplementary Figure 1 — Association of sNRP-1 with BRCA1/2 mutational status and CA125. (A) Scatter plots with sNRP-1 levels of patients with known BRCA1/2 mutational status are shown with BRCA1/2 mutations (mBRCA1/2, n =15) and wild-type BRCA1/2 status (wtBRCA1/2, n = 24), p = 0.97. (B) The correlation of sNRP-1 and log (CA125) is shown, using non-parametric Spearman correlation (n = 88, p = 0.04) with simple linear regression (red line). [file Presentation_1.pptx]
